# Genetic Relatedness Is Uncoupled from Fruit Color in Sour Cherry: Evidence from SSR, *S-RNase*, and Expression Profiling

**DOI:** 10.3390/plants15071069

**Published:** 2026-03-31

**Authors:** Attila Hegedűs, Péter Pfeiffer, Endre György Tóth, Júlia Halász

**Affiliations:** 1 Group of Horticultural Plant Genetics, Department of Plant Biotechnology, Institute of Genetics and Biotechnology, Hungarian University of Agriculture and Life Sciences, Ménesi út 44, 1118 Budapest, Hungary; hexaan@gmail.com (P.P.); julia.halasz@uni-mate.hu (J.H.); 2 Independent Researcher, 1118 Budapest, Hungary; 3 National Coalition of Independent Scholars (NCIS), Brattleboro, VT 05301, USA; endre.toth@ncis.org

**Keywords:** SSR markers, *S-RNase* genotyping, *Prunus cerasus*, fruit color variation, genetic diversity, population structure, anthocyanin biosynthesis, *MYB10* regulation

## Abstract

Sour cherry (*Prunus cerasus* L.) exhibits remarkable phenotypic and genetic diversity, historically classified into morello and amarelle groups based on fruit pigmentation. However, the genetic foundations of these categories remain unclear. Here, we combine 10 SSR loci with *S*-*RNase* genotyping to evaluate genetic diversity, phylogenetic relationships, and population structure across 27 Hungarian and internationally relevant sour cherry cultivars. The marker panel proved highly informative, yielding 78 SSR alleles and 17 *S*-alleles, with a multilocus probability of identity of 3.97 × 10^−7^. Phylogenetic reconstruction, minimum spanning networks, Bayesian clustering, and PCoA consistently resolved five genetically coherent groups that largely reflect known breeding histories and regional selection rather than fruit color classes. High- and low-anthocyanin cultivars frequently co-occurred within clades, demonstrating that pigmentation does not track genome-wide relatedness. To investigate proximate molecular mechanisms, we profiled flavonoid-pathway gene expression in contrasting accessions (VN-1 and ‘Pipacs 1’). VN-1 exhibited strong late-ripening induction of structural genes and *MYB10*, whereas ‘Pipacs 1’ showed attenuated late activation and higher early expression of *ANR*, *LAR*, and *UFGT*, suggesting divergent transcriptional regulation and pathway flux between the two genotypes. Together, these results indicate that fruit color variation is largely independent of the multilocus relatedness patterns captured by our marker set, and is likely influenced by lineage-specific regulatory differences.

## 1. Introduction

Sour cherry (*Prunus cerasus* L.) is a tetraploid (2*n* = 4× = 32) fruit tree of major economic and cultural significance across temperate regions, valued both for fresh consumption and for processing into juices, preserves, and nutraceutical products. Its origins are generally traced to natural hybridization between diploid sweet cherry (*Prunus avium*) and tetraploid ground cherry (*Prunus fruticosa*), with subsequent stabilization of the hybrid lineage [1,2]; this allotetraploid origin underpins the species’ extensive phenotypic and genetic diversity and presents distinctive challenges and opportunities for breeding and genetic analysis. Historically, two major cultivar groups have been recognized based on fruit pigmentation: the dark-fleshed, highly pigmented “morello” types and the lighter-red, less pigmented “amarelle” types, categories that remain useful descriptors of fruit quality [3,4].

Hungary occupies a prominent position in the secondary gene center of sour cherry and has long served as a source of distinctive cultivars and breeding materials [5,6,7]. Landmark Hungarian breeding and selection efforts initiated in the mid-20th century produced numerous commercially important cultivars and clonal selections (e.g., the Pándy and Cigánymeggy clones; later ‘Érdi bőtermő’, ‘Újfehértói fürtös’, ‘Kántorjánosi 3’), which contributed to the rapid expansion of sour cherry acreage and to diversification in fruit size, soluble solids, and juice color in Central and Eastern Europe [3]. In addition, ‘Újfehértói fürtös’ and ‘Érdi bőtermő’ were introduced into the United States in the 1980s by Dr. Amy Iezzoni (Michigan State University) and marketed under the name ‘Balaton’ and ‘Danube’, respectively, and used in breeding programs [8]. ‘Balaton’ became the second major sour cherry cultivar in North American production alongside ‘Montmorency’, expanding product portfolios for growers and processors. These examples illustrate how Hungarian selections have been deployed internationally as both cultivars for production and breeding parents or sources of adaptive traits, reinforcing Hungary’s role as a reservoir of useful alleles for fruit quality, maturity timing, and stress adaptation [9].

Simple sequence repeats (SSRs or microsatellites) have become powerful markers for genetic diversity studies. Owing to their high polymorphism, codominant inheritance, abundant distribution throughout the genome, and cross-transferability, they can be effectively applied across a wide range of *Prunus* species [10] for cultivar identification [11], germplasm characterization [12,13], and parentage analysis [14]. Like many *Prunus* species, sour cherry exhibits an *S*-RNase-based gametophytic self-incompatibility (GSI) system, in which the pistil determinant is an *S*-RNase and the pollen determinant an *S*-haplotype-specific F-box protein (SFB) [15]. Genotype-dependent loss of SI can arise through the accumulation of at least two non-functional *S*-haplotypes (mutations in *S-RNase* and/or *SFB*), a system named as ‘one allele-match model’ [16,17]. Because *S*-locus variation directly influences compatibility relationships and fruit set, *S*-genotyping has become a critical complement to neutral marker systems for breeding, orchard design, and germplasm characterization.

Sour cherry cultivars differ markedly in fruit anthocyanin content and composition, with phenotypes ranging from bright red (typical of ‘Montmorency’-type amarelle) to deeply pigmented, almost black morello types. This diversity has clear nutritional and functional implications and is of commercial interest to both fresh and processing sectors [4,18]. Anthocyanin accumulation derives from the phenylpropanoid/flavonoid pathway, in which flux through “late” biosynthetic steps (e.g., *DFR*, *ANS*, *UFGT*) is subject to coordinated transcriptional activation by R2R3-MYB transcription factors, bHLH proteins, and a WD40 scaffold (the canonical MBW complex) [19]. In cherries, *MYB10* orthologs have been identified as key positive regulators of skin coloration, with both cultivar- and allele-specific effects on the timing and intensity of pigmentation. Comprehensive studies on MYB-mediated control emphasize that temporal dynamics (developmental stage), tissue specificity, and environmental cues (light, temperature) modulate MBW activity, providing a flexible regulatory network capable of generating convergent pigmentation phenotypes across diverse genetic backgrounds [20,21,22].

The present work leverages a combined SSR + *S-RNase* marker set to (i) evaluate marker informativeness and discriminatory power in a panel of 27 representatives of Hungarian and internationally relevant sour cherry germplasm; (ii) reconstruct phylogenetic relationships and genetic structure; and (iii) assess whether fruit color classes (and by extension, historical morello/amarelle groupings) align with multilocus genetic relationships. We further integrate real-time PCR (qPCR) expression profiling of phenylpropanoid/flavonoid pathway genes—including *MYB10*—across ripening stages (RPS) in two contrasting cultivars to elucidate the mechanistic basis linking transcriptional regulation to pigmentation phenotypes.

## 2. Results

### 2.1. Informativeness of SSR and S-RNase Markers

Across the ten SSR loci and the *S-RNase* gene, the marker set captured broad allelic variation and strong discriminatory power (Table 1).

In 27 sour cherry genotypes, amplification of genomic DNA was successful in all the 10 SSR loci using primers designed for *Prunus* species (Table 1). Altogether, 10 primer pairs produced a total of 78 alleles ranging from 5 to 14 per locus. A maximum of four alleles per genotype were found in all loci according to the tetraploid genome of the species. In cases where fewer alleles were detected than expected, this could reflect either allele dosage effects or the presence of null alleles, which cannot be distinguished unambiguously in tetraploid SSR analyses. The range of fragment length was detected among the accessions from 102 to 213 bp, the mean value found was 7.8 alleles per locus. The *S-RNase* gene has 17 alleles in the tested sour cherries and hence proved to be even more polymorphic than any of the studied SSR loci.

Diversity metrics varied widely among loci, with the *S-RNase* marker consistently ranking at the top across effective allele number, polymorphic information content (PIC), Shannon’s index (*H*′), and resolving power (*Rp*). Among SSRs, BPPCT 037 and BPPCT 040 formed the highest-performing tier. In contrast, a small subset of loci (e.g., ASSR 17 and BPPCT 004), contributed comparatively little information. This pattern indicates that the panel combines highly powerful loci with a few moderate performers—an asset for balancing genome coverage with resolution.

Effective allele numbers spanned well over a sevenfold range, PIC exceeded a fivefold dynamic range, and *Rp* differed by more than an order of magnitude between the weakest and strongest loci. For the 11 combined marker loci, a PI of 3.97 × 10^−7^ (PD = 0.999) means that the probability of two unrelated individuals sharing the same multilocus genotype is approximately one in 2.5 million, indicating extremely high discriminatory power of the marker set. Collectively, Table 1 demonstrates that the marker suite provides ample allelic richness and resolution for both cultivar discrimination and population-level inference, supporting the use of the combined SSR and *S-RNase* dataset in downstream analyses.

### 2.2. Phylogenetic Analysis and Pairwise Genetic Distances

**Figure 1:**
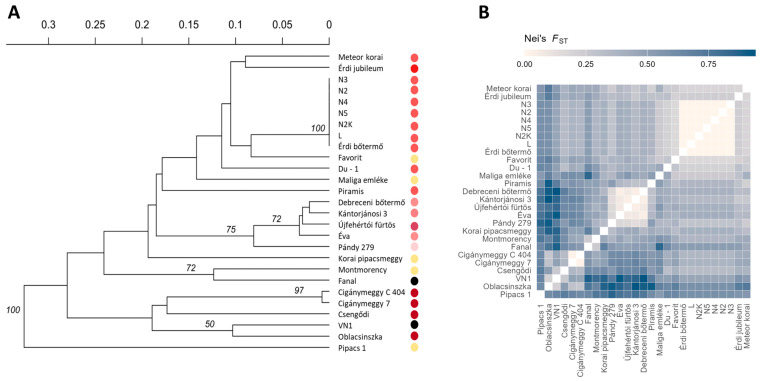
Genetic relationships among Hungarian sour cherry (*Prunus cerasus*) cultivars based on 10 simple sequence repeat (SSR) loci and *S-RNase* alleles. (**A**) The unweighted pair-group method with arithmetic mean dendrogram constructed in R illustrates genetic relationships among sour cherry cultivars. Similarity was assessed using Nei’s genetic distance. Node support values were estimated by bootstrap analysis with 10,000 replicates. (**B**) Heatmap of pairwise genetic differentiation (Nei’s *F*_ST_) among cultivars, showing relative divergence based on the combined SSR and *S-RNase* dataset.

Phylogenetic reconstruction and distance-based analyses revealed highly congruent patterns of genetic relationships among the genotypes of various origins. The UPGMA dendrogram resolved accessions into statistically supported clades of clones of ‘Érdi bőtermő’, the Northern Great Hungarian plain selections (‘Újfehértói fürtös’, ‘Kántorjánosi’, ‘Debreceni bőtermő’, ‘Éva’, ‘Petri’ and ‘Pándy 279’); and pairs of ‘Montmorency’ and ‘Fanal’, VN-1 and ‘Oblacsinszka’ and two Cigánymeggy clones (Figure 1A). Beyond identifying the major clades, the UPGMA dendrogram also reflects well known pedigree connections and regional breeding trajectories within the collection. The very short branch lengths observed within the ‘Érdi bőtermő’ complex correspond to their common origin and the minimal allelic differences detected among these accessions. Similarly, the compact cluster formed by cultivars from the Northern Great Hungarian Plain aligns with their shared selection history and previously reported SSR similarity patterns. The close grouping of VN-1 with ‘Oblacsinszka’, as well as the pairing of the Cigánymeggy clones, is consistent with their characteristic fruit traits, historical connections, and multilocus similarity profiles.

In contrast, cultivars with heterogeneous geographic origins—particularly ‘Pipacs 1’, ‘Fanal’ and ‘Montmorency’—form longer branches and cluster more distantly from other lineages, reflecting their elevated multilocus divergence and higher allelic richness, as also shown by within group diversity parameters (Table 2). Importantly, high and low anthocyanin cultivars appear across several clades rather than forming color specific clusters, indicating that fruit pigmentation does not follow the main axes of genome wide relatedness.

The heatmap of pairwise genetic differentiation highlighted clear contrasts between closely related and more divergent genotypes (Figure 1B) with ‘Pipacs 1’ showing pronounced differentiation from all other tested accessions.

**Figure 2:**
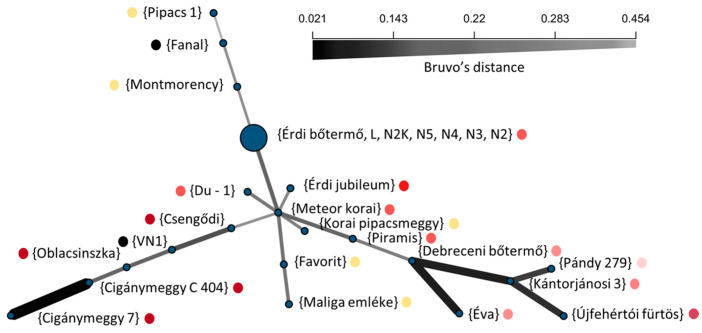
Minimum spanning network of Hungarian sour cherry (*Prunus cerasus*) cultivars, constructed from a combined multilocus distance matrix integrating the ploidy- independent Bruvo distance across 10 SSR loci with the Jaccard distance of the multiallelic, non-stepwise evolving *S-RNase* allele sets (treated as an additional independent locus). Colors indicate fruit anthocyanin content, ranging from yellow (low accumulation) to black (high accumulation).

The minimum spanning network (MSN) based on Bruvo’s distance further corroborated these relationships (Figure 2). Genotypes formed compact subnetworks corresponding to the major clades, whereas longer edges separated more distant lineages.

When fruit anthocyanin content was overlaid onto the network, contrasting groups were observed: while VN-1, ‘Oblacsinszka’, ‘Fanal’, and Cigánymeggy clones with high anthocyanin content grouped together, ‘Fanal’, ‘Montmorency’ and ‘Pipacs 1’ showed specific genetic neighborhoods while having a sharp contrast in anthocyanin content, suggesting no close relationships between multilocus genetic background and fruit color variation.

### 2.3. Population Structure Analysis

**Figure 3:**
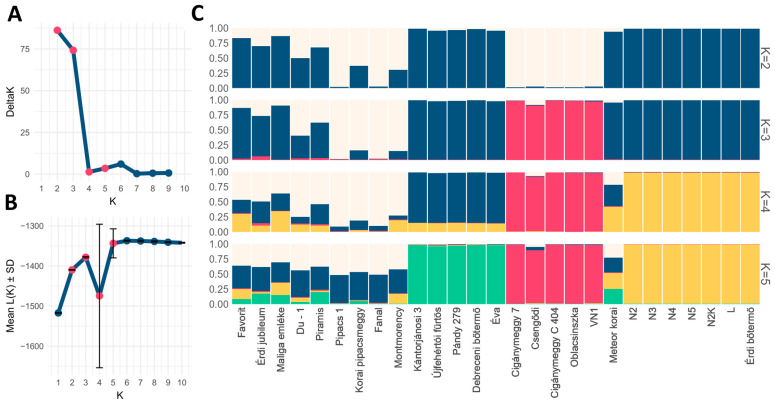
Bayesian clustering analysis of Hungarian sour cherry (*Prunus cerasus*) cultivars based on multilocus genotypes derived from 10 SSR loci and *S-RNase* alleles. Analyses included a burn-in of 100,000 iterations followed by 500,000 Markov chain Monte Carlo (MCMC) iterations. (**A**) ΔK values used to identify the optimal number of genetic clusters following the Evanno method. (**B**) Mean L(K) ± SD across replicated runs. (**C**) Admixture bar plot showing cultivar assignment probabilities for *K* = 2–5, averaged across runs using CLUMPP and visualized with the *pophelper* package in R. In the bar plot, K denotes the number of inferred genetic clusters, each represented by a different color.

Bayesian clustering analyses identified a clear optimum in the number of genetic clusters, supported by both ΔK profiles and likelihood values (Figure 3A,B). Although the Evanno method indicated an optimal K value of 2, this approach is known to detect only the uppermost hierarchical level of population structure. The ΔK analysis showed a clear peak at K = 2, indicating the highest hierarchical level of genetic structuring in the dataset. In contrast, the log-likelihood values [ln P(D|K)] increased and reached a plateau at K = 5, suggesting that this K value provided the most stable likelihood signal among the tested models.

**Figure 4:**
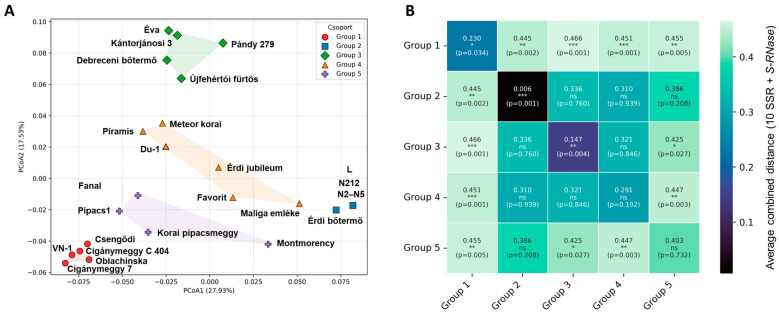
Analysis of genetic differentiation among the five predefined Hungarian sour cherry (*Prunus cerasus*) groups, calculated by combining the average Bruvo distance across 10 SSR loci with the Jaccard distance of *S-RNase* allele sets (weighted as an additional, 11th locus). (**A**) Principal Coordinate Analysis (PCoA). Coordinates were reconstructed from the inter-individual distance matrix using classical eigen decomposition, and the first two axes are shown, representing the largest proportions of explained variance. (**B**) Heatmap showing mean pairwise genetic distances among the five sour cherry groups. Diagonal cells display within-group mean distances; off-diagonal cells show between-group means. Each cell is annotated with the mean distance, significance level (*p*-value from 1999 permutation tests), and significance symbols (*** *p* ≤ 0.001, ** *p* ≤ 0.01, * *p* ≤ 0.05, ns > 0.05). Colors follow a black–turquoise scale, with lighter shades indicating greater genetic divergence.

Assignment plots revealed that most cultivars were strongly associated with a single cluster, although several showed admixed ancestries, indicating historical or ongoing gene flow among groups (Figure 3C). Principal coordinate analysis (PCoA) provided an independent multivariate confirmation of this structure (Figure 4A). The first two coordinates accounted for 27.9 and 17.5% of the total variation and separated the same clusters identified by the Bayesian approach, with within-cluster dispersion being markedly lower than between-cluster separation. This concordance across methods supports the robustness of the inferred genetic structure with 5 groups: (1) Dark color fruited genotypes (Cigánymeggy clones, ‘Oblacsinszka’, VN-1, and ‘Csengődi’); (2) ‘Érdi bőtermő’ and its clones; (3) selections in the north of Great Hungarian Plain of Hungary (‘Pándy 279’, ‘Újfehértói fürtös’, ‘Debreceni bőtermő’, ‘Kántorjánosi 3’ and ‘Éva’; as well as two admixed groups of (4) Pándy offspring (‘Meteor korai’, ‘Piramis’, ‘Érdi jubileum’, ‘Favorit’, ‘Maliga emléke’ and Du-1 selection with unknown origin) and (5) old cultivars with unknown ancestry (‘Montmorency’, ‘Fanal’, ‘Korai pipacsmeggy’, and ‘Pipacs 1’). As shown in Figure 4B, Groups 4 and 5 display lower internal compactness than the other groups, while Groups 1 and 5 exhibit greater separation from the remaining groups than the statistically non-significant distances among Groups 2, 3, and 4.

Within-group diversity parameters further distinguished the clusters (Table 2). Group 5 exhibited markedly higher allelic richness and a greater prevalence of unique variants, whereas Group 2 showed substantially reduced diversity. Differences among groups extended to the proportion of polymorphic loci, with some groups being polymorphic across all loci and others showing partial fixation. These contrasts are consistent with differing demographic histories and degrees of genetic exchange. Fruit pigmentation differed across the five groups, with two groups also displaying significant intra-group variation.

### 2.4. Differential Expression of Anthocyanin Biosynthetic Genes During Fruit Ripening

Across ripening, the two contrasting sour cherry genotypes examined in this study—VN-1, a dark-colored Hungarian selection with high anthocyanin accumulation, and ‘Pipacs 1’, a light-colored amarelle-type cultivar—both exhibited a general increase in fruit size, fresh weight, and soluble solids content, indicating coordinated physical growth and sugar accumulation during maturation (Tables S1 and S2). In parallel, skin color development followed a common trajectory characterized by a progressive decrease in lightness (L*) and hue angle (^o^), reflecting the transition from green/yellow to red coloration. The most pronounced difference between cultivars was observed at late RPS, where VN-1 fruits displayed a markedly stronger reduction in chroma (C*) and lightness. In contrast, ‘Pipacs 1’ fruits retained higher chroma and redness (a*), indicating a stable red coloration despite advanced ripening.

Given the pronounced divergence in skin color development between the two cultivars, we next examined the transcriptional dynamics of anthocyanin biosynthetic genes across RPS. The qPCR primers were designed based on homologous sequences retrieved from the NCBI GenBank database and the sour cherry genome sequence available at the Genome Database for Rosaceae (http://www.rosaceae.org/node/355, accessed on 20 January 2026). For several genes (*C4H*, *CHI*, *F3H*, *F3′5′H*, *LAR*, *ANR*, and *UFGT*), degenerate consensus primers (Table S3) were designed, and the resulting amplicons were sequenced from the cultivar ‘Pipacs 1’ (Table 3); the obtained sequences were used for primer verification. Expression analyses of the genes encoding cinnamate 3-hydroxylase (C3H) and 4-coumarate–CoA ligase (4CL) were performed using primer sequences previously reported by Dardick et al. [29]. Primer sequences used for real-time PCR and the expected amplicon sizes are listed in Table S4.

**Figure 5:**
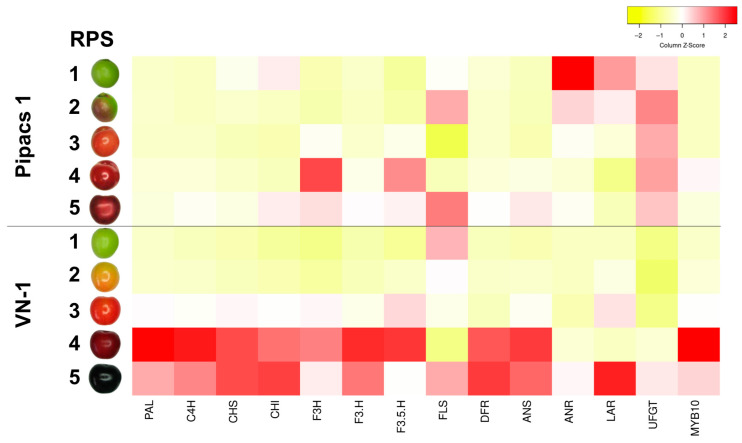
Heatmap of phenylpropanoid- and flavonoid-pathway gene expression in Hungarian sour cherry (*Prunus cerasus*) accessions ‘Pipacs 1’ and VN-1. Gene expression levels were quantified by qPCR using RP-II as reference gene and visualized as column Z-scores. The heatmap was generated with the Heatmapper online tool (http://heatmapper.ca, accessed on 1 February 2025), illustrating relative differences in transcript abundance across the analyzed genes.

Gene expression profiling of key components of the phenylpropanoid and flavonoid biosynthetic pathways revealed clear genotype-specific transcriptional patterns between the two sour cherry genotypes (Figure 5). Heatmap visualization of Z-score–standardized expression values demonstrated coordinated regulation of multiple pathway genes, indicating pathway-level transcriptional control rather than isolated gene-specific effects.

Comparative analysis showed broadly parallel expression trends across several biosynthetic genes in both cultivars, consistent with their contrasting anthocyanin accumulation phenotypes. In the high-anthocyanin-accumulating fruit peel of VN-1, transcript levels of the majority of phenylpropanoid and flavonoid pathway genes progressively increased during ripening, with pronounced induction observed at RPS 4 and/or 5. This coordinated upregulation was accompanied by a strong increase in the expression of the transcription factor *MYB10*, consistent with its proposed regulatory role in anthocyanin biosynthesis.

In the low-anthocyanin cultivar ‘Pipacs 1’, a similar temporal expression pattern was observed; however, the magnitude of transcriptional induction during the late RPS was substantially lower. Notably, *ANR*, *LAR*, and *UFGT* exhibited relatively higher expression levels at early ripening stages (RPS 1–2) in ‘Pipacs 1’ compared with VN-1, suggesting differences in pathway flux regulation during early fruit development.

## 3. Discussion

### 3.1. Efficiency and Resolution of SSR and S-RNase Markers in Tetraploid Sour Cherry

The combined panel of 10 SSR loci and the functional *S-RNase* marker performed with high analytical quality across all 27 accessions and matched the constraints of a tetraploid genome. Amplification succeeded at every locus, yielding 78 SSR alleles (5–14 per locus; mean 7.8) within a 102–213 bp range (Table 1), and 17 alleles at *S-RNase*, which was more polymorphic than any individual SSR locus (Table 1). As expected for tetraploids, up to four alleles were observed per genotype; instances with fewer than four are compatible with allele-dosage ambiguity and/or null alleles that cannot be unambiguously resolved with routine scoring in polyploids [31].

Diversity metrics consistently corroborated informativeness: *S-RNase* ranked at the top for *Ne*, PIC, *H*′, and *Rp*, while BPPCT 037, BPPCT 040 and CPSCT 021 formed a particularly strong SSR subset. Some loci show average polymorphism in diploid *Prunus* [32,33] but markedly higher polymorphism in polyploids [34,35], whereas markers such as ASSR 17 and BPPCT 004 contributed more modest information content. This intentionally mixed panel—combining highly and moderately informative loci—balanced genome coverage and discriminatory depth, minimized redundancy, and produced a robust multilocus signature for cultivar identification and population-level inference. The mean *Rp* of the SSRs (2.40) agrees with values reported from other *Prunus* assays using different loci [13,36] and increased substantially when *S-RNase* alleles were included. Relative to the guideline of Waits et al. [37] that multilocus probability of identity (PI) should be below 10^−4^ for reliable individual identification, our PI of 3.97 × 10^−7^ (PD = 0.999) indicates an ≈1 in 2.5 million chance that two unrelated individuals share the same multilocus genotype—adequate for cultivar authentication and pedigree reconstruction (Table 1).

Given the allele-dosage uncertainty in tetraploids, we emphasized allele counts, Shannon’s information index, and Bruvo’s distance rather than classical heterozygosity estimates. Although *S-RNase* is a functional locus that may deviate from strict neutrality, its high polymorphism augmented resolution without disrupting the broader relationship patterns, consistent with earlier reports [34,38].

From a practical standpoint, the panel’s performance has direct implications for germplasm management and breeding. First, the extremely high PD supports routine identity testing and mislabel detection in living collections. Second, the locus-by-locus performance profile (Table 1) provides a ranked shortlist (e.g., *S-RNase*, BPPCT 037, BPPCT 040) for reduced-panel genotyping when throughput or cost constraints apply. Third, the combined SSR + *S-RNase* dataset supplied sufficient allelic richness and resolution to support downstream phylogenetic and structure analyses. Looking ahead, integrating dosage-aware genotyping (e.g., SNPs or amplicon sequencing) would refine allele-copy estimates in tetraploids; nevertheless, for the scope of this study the present panel offers a cost-effective, high-confidence solution for both individual-level discrimination and population-level inference in sour cherry.

### 3.2. Phylogenetic Consistency and Genetic Structure Reflect Shared Breeding History Rather than Fruit Color

The complementary phylogenetic, network-based, and multivariate representations consistently identified the same major genetic groupings. Across methods, relationship and structure analyses suggested patterns broadly consistent with known pedigree connections and regional selection history. The UPGMA dendrogram resolved statistically supported clades consistent with known horticultural lineages [5,39], including Northern Great Hungarian Plain selections, the ‘Érdi bőtermő’ complex, pairs such as ‘Montmorency’–‘Fanal’, VN1–‘Oblacsinszka’, and Cigánymeggy clones (Figure 1A). Pairwise distances highlighted tight relatedness within these sets and consistently placed ‘Pipacs 1’ at larger distances (Figure 1B), explaining its outgroup position in the UPGMA tree. This pattern was mirrored by the MSN where short edges connected members of the same historical or geographic lineage (Figure 2).

Population structure showed a likelihood plateau at K = 5, and this value revealed biologically meaningful sub-structuring consistent with geographic origin and/or breeding history (Figure 3) [40]. PCoA offered orthogonal confirmation for separating the five clusters and showing tight within-cluster dispersion relative to between-cluster separation for at least three groups (Figure 4A). Group 1 collected cultivars and selections with dark colored, high anthocyanin containing fruit [4] with Cigánymeggy clones differing by only a single allele and ‘Oblacsinszka’/VN1 showing very few allelic differences. ‘Oblacsinszka’ and Cigánymeggy are considered autochtonous cultivars in Serbia and Hungary, respectively, with very similar traits like small fruit size, self-compatibility, acidic taste or high anthocyanin content [4,41] and SSR profiles [11]. Group 2 (‘Érdi bőtermő’ complex), formed a dense and well-delimited cluster of clones with only a minor difference at CPSCT 021, consistent with clonal propagation and focused selection within this group [42,43].

Group 3 comprised cultivars from the Northern Great Hungarian Plain, where Pándy clones likely arose [6]. According to historical accounts, self-compatible selections were already reported within the Pándy orchards in 1940 [44]. ‘Újfehértói fürtös’ shares the same *S*-haplotypes as Pándy clones except for a mutation in the *S*_1_′ allele [16], and the two differ at only six of 29 SSR alleles we detected. Their close relationship is further confirmed by other SSR loci [11].

Group 4 showed broader dispersion consistent with admixture (Figure 3C and Figure 4) and includes Pándy-derived hybrids, e.g., full siblings ‘Érdi jubileum’/‘Maliga emléke’ and the second-generation ‘Piramis’ [26]. Group 5 (old cultivars of unknown pedigree) exhibited elevated allelic richness and more private alleles (Table 2), indicative of a more diverse or reticulate origin. The diverse origins of ‘Montmorency’ (France), ‘Fanal’ (Germany) and ‘Pipacs 1’ (Hungary) [3] align with the observed elevated PA accumulation and allelic richness relative to more narrowly bred groups. Interestingly, ‘Korai pipacsmeggy’, a hybrid derived from Pándy, is separated from the other Pándy-related cultivars in Group 3, possibly due to its second parent ‘Császármeggy’, a historical cultivar that has since disappeared and for which no reliable description has been preserved.

‘Pipacs 1’, the accession most distant in the multilocus analysis, originates from Kecel, Hungary [3]. Its distinctiveness is supported by previous SSR analyses [45] and further highlighted by its unique ability to accumulate genistein-type isoflavonoids in the fruit, a feature not detected in any other sour cherry cultivar [46]. Its amarelle-type fruit resembles those of Pándy and ‘Montmorency’—both long cultivated in Hungary—and this similarity led to earlier hypotheses of a close relationship. However, Halász et al. [26] demonstrated that ‘Pipacs 1’ shares only a single *S*-haplotype (*S*_36_) with either cultivar, and even this haplotype occurs as different mutant variants in all three, likely precluding direct parent–offspring relationship. It carries *S*_36b2_ (seen otherwise only in Group 1) and *S*_26_, detected only in this cultivar, Cigánymeggy clones and ‘Oblacsinszka’ [26,47,48]. Based on this pattern, Halász et al. [26] proposed that the amarelle ‘Pipacs 1’ may descend from the morello-type Cigánymeggy, despite their contrasting anthocyanin profiles. Our SSR data does not contradict this possibility, as none of the 10 loci examined provided evidence against such a relationship.

Overall, while Group 1 contains high-anthocyanin cultivars, genetic partitions rarely align with color; dark and light genotypes occur together in clades and clusters (Figure 1, Figure 2, Figure 3 and Figure 4), exemplified by the non-co-segregation of ‘Fanal’ (dark morello) and ‘Pipacs 1’ (light amarelle). The major axes of structure therefore reflect shared breeding history and regional selection, not fruit color, motivating a regulatory explanation.

Methodologically, the congruence among tree-, network-, ordination- and model-based approaches supports the internal consistency of these approaches within the limits of the dataset (Figure 1, Figure 2, Figure 3 and Figure 4). ΔK is known to emphasize the highest hierarchical split, whereas likelihood plateaus can reveal substructure; interpreting both yielded a faithful representation of complexity. Patterns of admixture in several cultivars (Figure 3C) likely reflect historical germplasm exchange and introgression common in cherry breeding, explaining why some groups (e.g., Groups 4–5) are less compact (Figure 4B). Together, these findings provide an evolutionary and breeding context for the collection and suggest that fruit color is not a reliable indicator of the multilocus genetic groupings detected here.

### 3.3. Fruit Color Variation Is Uncoupled from Genome-Wide Genetic Relationships

Historically, sour cherries were classified as morello or amarelle by fruit/juice color [49], a scheme often treated as genetically meaningful. In our diverse Hungarian germplasm, however, color does not segregate with multilocus relatedness: dark and light genotypes occur together in UPGMA clades (Figure 1A), show short cross-phenotype distances (Figure 1B), and overlap in the MSN (Figure 2), while PCoA and STRUCTURE at K = 2 and K = 5 likewise place both color classes within common groups (Figure 3C and Figure 4A). Thus, fruit color is not a reliable indicator of genome-wide relatedness in sour cherry.

Reports from a narrowly related Finnish collection found amarelles and morellos to cluster separately using nine SSR primers and pointed to group-specific alleles [50]. In our broader material, the previously suggested type-specific variants were not diagnostic: the 180 bp allele at BPPCT 002 occurs in several amarelles (‘Montmorency’, ‘Pipacs 1’, ‘Fanal’, ‘Korai pipacsmeggy’), the 124 bp variant at BPPCT 038 is present in ‘Pipacs 1’, and the 131 bp variant at BPPCT 039 in ‘Favorit’ and ‘Du1’; similarly, the 131 bp allele at BPPCT 040 is not confined to morellos [50]. Together with contrasts such as the related network positions of ‘Fanal’ (dark) and ‘Pipacs 1’ (light), or the close relatedness yet opposing color types of ‘Újfehértói fürtös’ (morello) and ‘Pándy 279’ (amarelle) [4,18], these findings indicate that similar color phenotypes have evolved repeatedly on distinct genetic backgrounds, while closely related genotypes can diverge markedly in pigmentation (Table 2). Within-group diversity parameters (Table 2) further differentiate clusters chiefly by allelic richness and the incidence of private variants rather than by shared color class, reinforcing that pigmentation is a cultivar-level trait arising on multiple genetic backgrounds.

A possible mechanistic explanation emerges when considering these transcriptional differences, although confirmation across a broader germplasm set will be necessary. Changes in the color parameters L*, H^o^, C* and a* reflected the consistently red versus balck coloration of ‘Pipacs 1’ and VN-1 fruits. The expression heatmap (Figure 5) shows coordinated late-stage up-regulation of phenylpropanoid/flavonoid genes in VN1 black colored fruit skin with a strong increase in *MYB10*, consistent with robust pathway activation during ripening [51], and *MYB10* expression levels correlate with major anthocyanin contents in sweet cherry [52]. By contrast, ‘Pipacs 1’ displays attenuated late-stage induction but comparatively higher early *ANR*, *LAR* and *UFGT* expression (RPS 1–2). This elevated early expression of these genes, despite the low *MYB10* transcript levels, is consistent with the possibility that additional MYBs (e.g., MYB15, MYBPA1) and potentially other transcription factors such as WRKY or NAC [53,54,55] contribute to the early-stage regulation of flavonoid biosynthesis, although further genotypes will be required to test this hypothesis.

These patterns implicate differences in transcriptional timing and intensity—rather than deep phylogenetic splits—as primary determinants of color outcomes. Regulatory divergence at key control nodes (e.g., target genes or their regulatory networks) provides a parsimonious route to convergent dark coloration on different genomic backgrounds or divergent color outcomes among closely related genotypes [8,56].

### 3.4. Transcriptional Regulation as the Proximate Driver of Color Divergence: Implications and Limitations

Expression profiling integrates the phenotypic and genetic results. VN-1 shows concerted late-ripening induction of multiple pathway genes with pronounced MYB10 up-regulation (Figure 5), consistent with activation of anthocyanin biosynthesis during terminal stages of ripening [51,57,58]. ‘Pipacs 1’, in contrast, exhibits weaker late induction and relatively higher early *ANR*/*LAR*/*UFGT* expression (RPS 1–2), aligning with metabolite data: ripe ‘Pipacs 1’ fruit contain ~7× more catechin and ~8× more procyanidin than VN-1 [59] and 2–3× more phenolic acids than high-anthocyanin cultivars [18], consistent with precursor diversion away from anthocyanin synthesis. Phenotypically, VN-1 darkens strongly and loses chroma at full ripeness, while ‘Pipacs 1’ retains a stable bright-red hue at comparable stages (Tables S1 and S2). Two non-exclusive scenarios follow: (i) convergent dark pigmentation can arise on unrelated genomic backgrounds when regulatory states are similar (e.g., strong late *MYB10* and activation of “late” genes); (ii) closely related genotypes may diverge in color when the timing or intensity of transcriptional programs differs [60,61]. Similar regulatory shifts appear in other *Prunus* species as well [62,63].

For breeding, regulation-aware strategies should be prioritized: include regulatory markers such as *MYB10*; assay stage-specific expression of late pathway genes as functional markers; and preserve regulatory haplotype diversity in germplasm management. These can be implemented alongside the reduced SSR + *S-RNase* subsets for identity testing and pedigree checks. Functional validation—promoter haplotyping, eQTL mapping and transient assays—will help link transcriptional dynamics to pigmentation phenotypes and deliver deployable markers.

Several caveats temper our conclusions. The neutral marker panel samples a small genomic fraction and causative color loci/regulatory regions may be unlinked to our markers; environmental and developmental factors (light, temperature, ripening cues) may also add variance [64,65]; the qPCR panel targeted only a subset of structural genes plus *MYB10*, leaving additional bHLH/WD40 cofactors and promoter variants unassayed [66,67]. Because gene-expression profiling was performed on only two contrasting genotypes, the regulatory interpretations presented here should be viewed as genotype-specific observations rather than species-wide generalizations. Future work including additional cultivars will be required to evaluate the broader relevance of these transcriptional patterns. Post-transcriptional/translational regulation, transport/sequestration (tonoplast transporters, GST-mediated targeting) and glycosylation/acylation can modulate visible pigmentation [55]; and tetraploidy introduces allele-dosage and homeolog-specific expression effects that can obscure simple marker–phenotype associations. Despite these caveats, the agreement among relationship methods and the alignment with expression profiles support the central conclusion that fruit color is largely decoupled from multilocus relatedness and is governed by differences in transcriptional regulation.

## 4. Materials and Methods

### 4.1. Sample Collection and Phenotypic Assessment

Leaf samples were collected from all cultivars and selections listed in Figure 1 at the University of Debrecen Research and Extension Centre for Fruit Growing (Újfehértó, Hungary). Two sour cherry genotypes (VN-1 and ‘Pipacs 1’) were sampled at five fruit developmental stages, ranging from fully immature to fully ripe. Fruit size, fresh weight, and color parameters were recorded on 35 replicates, while total soluble solids (°Brix) were measured on 5 replicates, using digital calipers, a gravimetric balance, a Konica Minolta CR-410 colorimeter (Konica Minolta, Inc., Tokyo, Japan), and a digital refractometer (ATAGO Corporation, Tokyo, Japan), respectively. Leaf and fruit skin samples were immediately frozen in liquid nitrogen (Linde Gas, Budapest, Hungary) and stored at −80 °C.

### 4.2. DNA Extraction and Simple Sequence Repeat Analysis

Genomic DNA was isolated from young leaves using the DNeasy Plant Mini Kit (Qiagen, Hilden, Germany). DNA quantity and quality were determined using a Nanodrop ND-1000 Spectrophotometer (Bio-Science, Budapest, Hungary). Ten previously published SSR primer pairs developed for *Prunus* species were used for genotyping: CPSCT 012, CPSCT 021 (Japanese plum; [25]), BPPCT 002, BPPCT 004, BPPCT 037, BPPCT 038, BPPCT 039, BPPCT 040 (peach; [24]), ASSR 17 and ASSR 63 (almond; [23]). The forward primers were labelled with 6-FAM fluorescent dye. PCR reactions were carried out in a PTC 200 thermocycler (MJ Research, Waltham, MA, USA) using the program described for the primers. Approximately 20–80 ng of genomic DNA was used for PCR amplification in a 25 μL reaction volume containing 10 × Dream*Taq*™ Green buffer (Fermentas, Szeged, Hungary) as well as KCl and (NH_4_)_2_SO_4_ at a ratio optimized for robust performance of Dream*Taq*™ DNA Polymerase in PCR with final concentrations of 4.5 mM MgCl_2_, 0.2 mM of dNTPs, 0.2 μM of the adequate primers and 0.75 U of Dream*Taq*™ DNA polymerase (Fermentas).

### 4.3. Allele Sizing and Data Analysis

Amplification success was verified on 1.2% TAE agarose gels, and DNA bands were visualized by ethidium bromide staining and compared with the 1 kb DNA ladder (Promega, Madison, WI, USA). Allele sizes were determined using an automated sequencer ABI Prism 3100 Genetic Analyzer (Applied Biosystems, Budapest, Hungary), GENOTYPER 3.7 software, and the GS500 LIZ size standard (Applied Biosystems). Published *S*-genotypes [26] were incorporated in the dataset for diversity and phylogenetic analysis.

### 4.4. Genetic Diversity and Population Structure Analyses

Genetic diversity indices were computed per locus in MS Excel as follows: *Na* (number of alleles), *Ne* (effective number of alleles, *Ne* = 1/Σ*p_i_*^2^), PIC (polymorphic information content estimated from allele frequencies following the Botstein method [27]), *H*′ (Shannon’s information index using natural logarithms, *H*′ = −Σ*p_i_* ln *p_i_* [68]), PA (private alleles; alleles occurring in only one accession), and *Rp* (resolving power; *Rp* = Σ*I_b_*, where *I_b_* = 1 − 2 |0.5 − *p*|, with *p* the proportion of accessions carrying the allele [69].

Genetic relationships on a combined SSR + *S-RNase* genetic distance matrix were assessed using complementary approaches. An unweighted pair group method with arithmetic mean (UPGMA) dendrogram was constructed based on Nei’s genetic distances [70,71] using 10,000 bootstrap replicates, as implemented in the poppr package [72] in R [73]. Only branches with bootstrap support ≥ 40% were retained. Pairwise Nei’s genetic distances (Nei’s pairwise *F*_ST_ [71]) were computed using the poppr distance function, which is suitable for polyploid datasets, and the resulting distance matrices were displayed as heatmaps.

Minimum Spanning Networks (MSN) were generated from a multilocus distance matrix that integrated the ploidy-independent Bruvo distance for SSR loci with the Jaccard distance of the multiallelic, non-stepwise evolving *S*-*RNase* allele sets (the *S*-locus treated as an additional independent marker) [74,75], and visualized using the interactive imsn tool in poppr. Principal coordinate analysis (PCoA) was performed on the same combined multilocus genetic distance matrix described above. Classical eigen-decomposition [76] was used to obtain principal coordinate axes. Group separation and within-group compactness were evaluated by permutation testing (1999 permutations) and matplotlib was used for generating high-resolution graphical output [77].

To infer population structure, a model-based Bayesian clustering analysis was conducted in STRUCTURE v.2.3.4 [78] under an admixture model with correlated allele frequencies. Twenty independent runs were performed for each K value, the estimated number of genetic clusters, ranging from 1 to 10, each with a burn-in period of 100,000 iterations followed by 500,000 MCMC iterations. Runs were averaged using CLUMPP v.1.1.2 [79]. The optimal K, the maximal value of the posterior probability, was determined using the Evanno method [80], based on ΔK and mean L(K) values, and visualized as bar plots as implemented in the pophelper package [81] in R.

### 4.5. RNA Extraction and cDNA Synthesis

Total RNA was isolated from approximately 200 mg of fruit skin tissue using the hot borate extraction method [82] and treated with DNase I (Fermentas Life Sciences, Burlington, ON, Canada) to remove genomic DNA. RNA concentration and purity were assessed using a NanoDrop ND-1000 spectrophotometer (NanoDrop Technologies, Wilmington, DE, USA). cDNA synthesis was carried out using the RevertAid™ First Strand cDNA Synthesis Kit (Fermentas) with 1 µg of total RNA and oligo(dT)18 or random hexamer primers. Reactions contained 20 U RiboLock RNase inhibitor and 200 U M-MuLV reverse transcriptase. Approximately 50 ng cDNA was used per PCR and qPCR reaction.

### 4.6. Primer Design and Gene Amplification

Primers targeting key phenylpropanoid and flavonoid pathway genes (*PAL, C4H, 4CL, CHS, CHI, F3H, F3′H, F3′5′H*, *DFR*, *ANS*, *ANR*, *LAR*, *UFGT*, *FLS*, *GAPDH*) were designed based on *Prunus* sequences retrieved from NCBI GenBank. For *PAL*, previously published primers were used [83]. Primer sequences, annealing temperatures, and expected amplicon sizes are listed in Table S1. PCR reactions were performed with 50 ng genomic DNA or cDNA in 25 µL volumes containing reaction buffer, 2.5 mM MgCl_2_, 0.2 mM dNTPs, 0.3 µM of each primer, and 1 U Taq DNA polymerase (Fermentas). Thermal cycling consisted of 95 °C for 2 min, followed by 35 cycles of 95 °C for 30 s, 53–60 °C for 1 min, and 72 °C for 1 min, with a final extension at 72 °C for 5 min.

### 4.7. Cloning and Sequencing

PCR products were gel-purified using the QIAquick Gel Extraction Kit (Qiagen) and cloned into the pGEM-T Easy vector (Promega). Recombinant plasmids were transformed into *E. coli* JM109 competent cells and screened by colony PCR using M13 universal primers. Plasmid DNA was isolated using a Rapid Plasmid DNA Mini-Prep Kit (Bio Basic, Markham, ON, Canada) and sequenced bidirectionally on an ABI PRISM 3100 Genetic Analyzer (Applied Biosystems). For each gene, three independent clones were sequenced, and sequences were deposited in the NCBI GenBank database.

### 4.8. qPCR Analysis

Gene expression analyses were performed using a Rotor-Gene 6000 real-time PCR system (Qiagen) with EvaGreen^®^ dye. Reactions were carried out in 20 µL volumes containing MyTaq™ HS Master Mix, EvaGreen^®^, 0.6 µM of each gene-specific primer, and 50 ng cDNA. All reactions were run in three biological replicates. The qPCR cycling program consisted of 95 °C for 2 min, followed by 40 cycles of 95 °C for 8 s and 60 °C for 8 s. Melt curve analysis (70–95 °C, 0.25 °C increments) confirmed the specificity of all amplifications.

Six reference gene primers were evaluated for expression stability: three previously published (18S rRNA, UBQ10, TEF II; [84]) and three newly designed (ACT, GAPDH, RP II). RP II exhibited the greatest stability across fruit developmental stages and was used for normalization. Relative gene expression levels were quantified using gene-specific primers designed for sour cherry (Table S2) and REST© (Relative Expression Software Tool, version 1.0). Heatmaps were generated using the Heatmapper online tool (http://heatmapper.ca, accessed on 1 February 2025).

## 5. Conclusions

Our findings show that fruit color variation in sour cherry does not show a consistent association with the multilocus genetic structure observed in this study. The combined SSR and *S-RNase* marker set proved highly informative for cultivar discrimination and resolved five genetically coherent groups shaped largely by breeding history and regional selection. Dark- and light-fruited cultivars frequently appeared within the same clusters, demonstrating that pigmentation traits are not suitable indicators of lineage. Gene-expression profiling of the two contrasting genotypes supports the idea that color differences may be associated with variation in the timing and strength of flavonoid pathway activation. Together, these results clarify the structure of Hungarian and internationally relevant germplasm, identify efficient markers for genetic identification, and highlight transcriptional regulation—rather than ancestry—as the main driver of color divergence. These insights provide a valuable framework for germplasm management and for developing breeding strategies that incorporate regulatory and functional markers.

## Figures and Tables

**Table 1:** Characteristics and genetic diversity parameters of ten SSR loci and the *S-RNase* gene in *Prunus cerasus* (27 genotypes), including allele number, effective allele number, private alleles, PIC values, Shannon’s diversity index, and resolving power. Primer characteristics (annealing temperature, amplicon size range, and original references) are also provided for each locus, together with locus-wise means.

Primer	LG	Ta (°C)	Range Size (bp)	Reference	*Na*	*Ne*	PA	PIC	*H* *′*	*Rp*
ASSR 17	5	60	186–213	[23]	5	1.424	3	0.298	0.673	0.519
ASSR 63	8	55	150–171	[23]	5	2.562	1	0.610	1.18	2.593
BPPCT 002	2	57	166–184	[24]	5	3.703	1	0.730	1.379	1.852
BPPCT 004	2	57	158–196	[24]	5	1.835	1	0.455	0.924	0.593
BPPCT 037	5	57	126–164	[24]	14	5.531	4	0.819	2.027	4.222
BPPCT 038	5	57	102–134	[24]	11	4.366	5	0.771	1.711	2.296
BPPCT 039	3	57	126–148	[24]	7	2.576	0	0.612	1.322	2.148
BPPCT 040	4	57	121–145	[24]	8	4.915	1	0.797	1.789	3.704
CPSCT 012	6	62	151–171	[25]	7	4.694	1	0.787	1.668	2.815
CPSCT 021	2	46	134–150	[25]	11	4.848	3	0.794	1.871	3.259
*S-RNase*	6	–	–	[26]	17	10.391	3	0.904	2.525	7.111
Mean		–	–	–	10.6	5.140	2.7	0.727	1.788	3.441

Note: Ta, annealing temperature; *Na*, number of alleles; *Ne*, effective number of alleles (*Ne =* 1*/Σpᵢ*^2^); PA, private alleles (alleles occurring in only one accession); PIC, polymorphic information content calculated using the Botstein et al. [27] formula; *H*′ (ln-based), Shannon’s information index calculated using natural logarithms; *Rp*, resolving power (*Rp = ΣIb*, where *Ib =* 1 *−* 2|0.5 −* p*|); bp, base pairs.

**Table 2:** Summary of within-group allelic diversity across five sour cherry (*Prunus cerasus*) groups, including the number of accessions, total allele number, average allele number per locus, private alleles, average number of private alleles per locus, and percentage of polymorphic alleles.

	Group 1	Group 2	Group 3	Group 4	Group 5
Number of accessions	5	7	5	5	5
*Na*	54	33	40	55	83
Avg. allele no.	4.91	3	3.64	5.00	7.55
PA	15	2	3	3	26
Avg. private alleles	1.36	0.18	0.27	0.27	2.36
Polymorphic alleles (%)	100	81.82	81.82	90.91	100
Fruit color (l/i/d)	0/0/5	0/5/0	1/4/0	0/6/0	3/0/1

Note: *Na*, number of alleles; Avg. allele no., average number of alleles per locus; PA, private alleles (alleles occurring in only one accession); Avg. private alleles, average number of private alleles per locus; Polymorphic alleles (%), proportion of loci exhibiting polymorphism within each group. Groups (G1–G5) correspond to the cultivar clusters defined in the main text; l/i/d indicate light, intermittent and dark red color of fruit according to Papp et al. [4] and Apostol [28].

**Table 3:** GenBank accession number, size and chromosomal location of partial mRNA sequences of flavonoid biosynthesis genes identified in ‘Pipacs 1’ sour cherry (*Prunus cerasus*), their closest homologs based on BLASTn analysis, associated accession numbers, homology significance (*E*-values).

Gene	Accession Number	Size (bp)	LG	Closest Homolog (BLASTn Hit, Accession Number, *E*-Value)
*C4H*	JQ622242	638	6A	HM204478.1 (*P. cerasifera* × *P. munsoniana*) *C4H*, *E* = 0
*4CL*	JQ622243	397	3A	XM_021946313.1 (*P. avium*) *4CL*, *E* = 0
*CHI*	JQ622244	441	2A	XM_034347094.1 (*P. dulcis*) *CHI*, *E* = 0
*F3H*	JQ622245	832	7	KP347503.1 (*P. avium*) *F3H*, *E* = 0
*F3′5′H*	JQ622246	335	5B	XM_021954639.1 (*P. avium*) *F3′5′H* EST, *E* = 10^−173^
*ANR*	JQ622247	804	4A	XM_021972616.1 (*P. avium*) *ANR*, *E* = 0
*LAR*	JQ622248	352	1	XM_008244943.1 (*P. mume*) *LAR*, *E* = 10^−174^
*UFGT*	JQ622249	371	3A	XM_021970907.1 (*P. avium*) *UFGT*, *E* = 10^−169^

Note: *C4H*, *cinnamate 4-hydroxylase*; *4CL*, *4-coumarate-CoA ligase*; *CHI*, *chalcone isomerase*; *F3H, flavanone 3-hydroxylase*; *F3′5′H, flavonoid 3′,5′-hydroxylase; ANR*, *anthocyanidin reductase*; *LAR*, *leucoanthocyanidin reductase*; *UFGT*, *UDP-glucose:flavonoid 3-O-glucosyltransferase*. LG, genomic positions according to the *P. cerasus* ‘Montmorency’ v1.0 genome assembly [30].

## Data Availability

Due to privacy issues, the data presented in this study are available on reasonable request from the corresponding author.

## Supplementary Materials

The following supporting information can be downloaded at https://www.mdpi.com/article/10.3390/plants15071069/s1, Table S1: Changes in fruit size, weight, soluble solids content, and skin color parameters during fruit ripening stages 1-5 of VN-1 (Group 1) sour cherry (*Prunus cerasus*); Table S2: Changes in fruit size, weight, soluble solids content, and skin color parameters during fruit ripening stages 1-5 of ‘Pipacs 1’ (Group 5)sour cherry (*Prunus cerasus*); Table S3: Nucleotide sequences, annealing temperatures, and expected amplicon sizes of primers used for PCR amplification of flavonoid biosynthetic genes in sour cherry (*Prunus cerasus*); Table S4: Nucleotide sequences and amplicon sizes of gene-specific primers used for quantitative real-time PCR (qPCR) analysis of the flavonoid pathway-related genes in *Prunus cerasus* fruit.

## Abbreviations

The following abbreviations are used in this manuscript:

4CL	4-Coumarate-CoA ligase
ANR	Anthocyanidin reductase
ANS	Anthocyanidin synthase
bp	Base pair
C3H	Cinnamate 3-hydroxylase
C4H	Cinnamate 4-hydroxylase
CHI	Chalcone isomerase
CHS	Chalcone synthase
DFR	Dihydroflavonol 4-reductase
F3H	Flavanone 3-hydroxylase
F3′5′H	Flavonoid 3′,5′-hydroxylase
FLS	Flavonol synthase
GAPDH	Glyceraldehyde-3-phosphate dehydrogenase
GSI	Gametophytic self-incompatibility
*H’*	Shannon index
K	Number of clusters
*L**	Lightness parameter
LAR	Leucoanthocyanidin reductase
MCMC	Markov chain Monte Carlo
MSN	Minimum spanning network
*MYB10*	Transcription factor
*Na*	Number of alleles
*Ne*	Effective number of alleles
PA	Private alleles
PCoA	Principal coordinate analysis
PIC	Polymorphic information content
PI	Probability of identity
qPCR	Quantitative real-time PCR
*Rp*	Resolving power
RPS	Ripening stage
*S*-RNase	Stylar ribonuclease
SFB	S-haplotype-specific F-box protein
SSR	Simple sequence repeat
TSS	Total soluble solids
UFGT	UDP-glucose:flavonoid 3-O-glucosyltransferase
UPGMA	Unweighted pair-group method with arithmetic mean
