# Supplementary material for: Genetic Relatedness Is Uncoupled from Fruit Color in Sour Cherry: Evidence from SSR, S-RNase, and Expression Profiling
Source: Plants (Basel). 2026 Mar 31;15(7):1069. doi: 10.3390/plants15071069 (PMC13075033; doi:10.3390/plants15071069)
Supplement: Supplementary file 1 [file plants-15-01069-s001.zip › plants-4184194-supplementary.pdf]

Table S1. Changes in fruit size, weight, soluble solids content, and skin color parameters during fruit ripening stages 1-5 of VN-1 (Group 1) sour cherry (*Prunus cerasus*).

	**Ripening stages**				
	**1**	**2**	**3**	**4**	**5**
Height (mm)	15.1±0.9	15.4±0.6	16.5±0.4	15.9±0.8	16.9±0.6
D_{VD} (mm)	14.3±1.0	15.1±0.8	16.2±0.3	15.9±0.6	16.9±0.7
D_L (mm)	15.2±1.8	16.9±0.4	18.7±0.7	18.6±0.8	19.8±0.8
Weight (g)	2.2±0.2	2.8±0.3	3.2±0.3	3.2±0.4	4.1±0.4
SSC	7.86±0.05	8.25±0.37	9.35±0.46	10.38±1.05	22.05±1.59
$L*$	73.9±1.7	55.9±3.5	35.5±4.3	35.2±3.1	27.8±0.6
$a*$	-10.3±3.3	28.9±5.4	34.5±6.0	29.1±5.2	4.2±1.2
$b*$	42.8±3.1	28.9±2.1	16.5±5.0	13.7±4.3	2.6±0.4
$C*$	44.1±3.3	41.2±3.6	38.3±7.5	32.2±6.5	4.9±1.2
H^o	103.4±4.1	45.4±6.5	24.9±3.4	24.7±3.0	32.6±5.8

Note: Fruit height, transverse diameters measured in the ventral–dorsal (D_{VD}) and lateral (D_L) directions, fresh weight, soluble solids content (SSC), and skin color parameters ($L*$, $a*$, $b*$) were determined at each ripening stage. Chroma ($C*$) and hue angle (H^o) were calculated from $a*$ and $b*$ values. Data are presented as mean ± SD.

Table S2. Changes in fruit size, weight, soluble solids content, and skin color parameters during fruit ripening stages 1-5 of 'Pipacs 1' (Group 5)sour cherry (*Prunus cerasus*).

	**Ripening stages**				
	**1**	**2**	**3**	**4**	**5**
Height (mm)	13.3±0.2	13.2±0.6	13.3±0.6	17.0±0.8	18.3±0.8
D_{VD} (mm)	12.6±0.4	12.4±0.6	12.9±0.7	17.5±0.9	19.1±1.1
D_L (mm)	11.9±0.5	11.6±0.6	12.1±0.8	19.4±1.2	21.6±1.2
Weight (g)	1.0±1.5	1.1±0.1	1.3±0.2	3.8±0.6	5.6±0.8
SSC	14.83±0.4	18.5±0.88	20.85±1.05	18.9±0.73	18.58±0.94
L^*	65.2±5.5	65.2±5.5	39.2±4.6	24.9±3.3	31.4±1.3
a^*	-19.6±3.1	-19.6±3.1	18.1±4.4	25.3±3.6	23.9±3.5
b^*	39.6±4.9	39.6±4.9	15.8±2.5	11.9±1.9	9.6±1.9
C^*	44.2±5.5	44.2±5.5	24.5±2.8	27.9±3.9	25.8±3.9
H^o	116.3±2.4	116.3±2.4	41.7±9.8	25.2±2.2	21.8±1.6

Note: Fruit height, transverse diameters measured in the ventral–dorsal (D_{VD}) and lateral (D_L) directions, fresh weight, soluble solids content (SSC), and skin color parameters (L^*, a^*, b^*) were determined at each ripening stage. Chroma (C^*) and hue angle (H^o) were calculated from a^* and b^* values. Data are presented as mean ± SD.

Table S3. Nucleotide sequences, annealing temperatures, and expected amplicon sizes of primers used for PCR amplification of flavonoid biosynthetic genes in sour cherry (*Prunus cerasus*)

Primer name	Primer sequence	T_a (°C)	Amplicon size (bp)
C4H-F1	CTTCACBAACAARGTSGTSC	56.5	678
C4H-R1	CYTTSAYCACRGCTTGVAG		
4CL-F1	GAGATTTGCATTAKAGGTGACCA	57	442
4CL-R1	GCTMTCAASTCCTTCCGCAAGAT		
CHI-F	GAARGGYAAGACSGCCSAGGAG	57.5	481
CHI-R	TCSAMTTTYYCATTTCCGGC		
F3H-F	GAWATYCCGATCATYTCSCT	53	874
F3H-R	TCATCTTCTTCTTGTACATCTC		
F3'5'H-F1	TTACAAGCTGTGATGAAAGAAAC	55.5	380
F3'5'H-R1	CCAAACTTCTCWGAAAGATCCA		
ANR-F	GAAACTTAGCAACTGAAAGAGC	55	846
ANR-R	GCTCAGGAACACTGGTATTG		
LAR-F3	CCTTCTAAGGCTGACAYCRTCAA	58	395
LAR-R2	GGTGGVABAACCTCVGAAGG		
UFGT-F4	CCDCCGKTGTAYCCKGTGG	60	410
UFGT-R1	TTCCACCCRCARTGRGABAC		

PAL primers were previously designed for cherry and described by Sonneveld et al. [83].

Table S4. Nucleotide sequences and amplicon sizes of gene-specific primers used for quantitative real-time PCR (qPCR) analysis of the flavonoid pathway-related genes in *Prunus cerasus* fruit.

**Primer**	**Primer sequence**	**Amplicon size (bp)**
qRP-II-F3	CATGCCAAGTGGTCACCTGCAG	126
qPR-II-R3	GGTAGGACTACTTTCAACCCAAGCCTTC	
qPAL-F2	GCTAAGAAGTTGCACGAGCAGGA	115
qPAL-R2	TGGTGGAGTACCGGATCACTTCG	
qC4H-F1	GGCGGTTGCAGCTGATGATGTAC	191
qC4H-R1	CAAGTAGCCTCTCAAGAAGGGTCTC	
q4CL-F2*	CCAACTCACCTACGCTCAACTCTG	127
CL-R2*	CACCACTGGGAAGAAGATGGAG	
qHCT-F1	CTGGTTACTTTGGCAATGTGATTTTCAC	172
qHCT-R1	GACAGATCAGGCTGAAGCTCAAG	
qC3H-F*	TCAGACTACTTCCGTTTGGAGCAG	166
qC3H-R*	ACAAGCCCTGGATTTTCCGAC	
qCHS-F2	AGTGGTGTGGACATGCCTGG	133
qCHS-R2	AGTCCTTGGCCAACCGGAG	
qCHI-F1	CCACTAACCGGCCAGCAATA	104
qCHI-R1	TTCTCAATGGCCTTGGCTTC	
qF3H-F2	GTGGATCACCGTTCAACCAGTGG	146
qF3H-R2	GAATGTGGCTATGGACAGCCTGC	
qF3’H-F1	GACACGTCATCAAGCACAGTGGA	212
qF3’H-R1	GCCATGCGAGGCAACGAG	
qF3’5’H-F1	GTGGGAGGCTACACCATTCC	213
qF3’5’H-R1	CACCATCCTCTCAGCCATTGC	
qDFR-F3	TTGAATTTTGCCGCTCTGTCAA	143
qDFR-R3	AAATGGGCCAATCACAAGAGTTG	
qFLS-F1	GTGGTGGCTCACACCGACATGTC	121
qFLS-R1	TGACAAGGGCATTAGGGATGTAC	
qANS-F2	ATGCAGGGAGGAGTTGAAGAAGG	133
qANS-R2	TTGCTCAATGGGAAGATCGAAAA	
qANR-F1	CCACCCAACCCATCTCAAAG	102
qANR-R1	TGACGGCATAGCCCTTCTCT	
qLAR-F3	CCTCTTGACCCTTCTAAGGCTGAC	102
qLAR-R2	CAGTTTCTCCATCAGTGCCTTATCAG	
qUFGT-M-F1	GCTTTGGTGAGGACCAGGTG	156
qUFGT-M-R1	CGAGGAACCCTTCAGGCAAG	
qMYB10-F1	GGAAGAGCTGTAGACTAAGGTGGTTG	132
qMYB10-R1	GAGCAATCAATGACCACCTGTTTCC	

* Primers were originally reported by Dardick et al. [29] and used in this study.
